# An electromagnetic modulator based on electrically controllable metamaterial analogue to electromagnetically induced transparency

**DOI:** 10.1038/srep40441

**Published:** 2017-01-16

**Authors:** Yuancheng Fan, Tong Qiao, Fuli Zhang, Quanhong Fu, Jiajia Dong, Botao Kong, Hongqiang Li

**Affiliations:** 1 Key Laboratory of Space Applied Physics and Chemistry, Ministry of Education and Department of Applied Physics, School of Science, Northwestern Polytechnical University, Xi’an 710129, China; 2 The Institute of Dongguan-Tongji University, Dongguan 523808, Guangdong, China

## Abstract

Electromagnetically induced transparency (EIT) is a promising technology for the enhancement of light-matter interactions, and recent demonstrations of the EIT analogue realized in artificial micro-structured medium have remarkably reduced the extreme requirement for experimental observation of EIT spectrum. In this paper, we propose to electrically control the EIT-like spectrum in a metamaterial as an electromagnetic modulator. A diode acting as a tunable resistor is loaded in the gap of paired wires to inductively tune the magnetic resonance, which induces remarkable modulation on the EIT-like spectrum through the metamaterial sample. The experimental measurements confirmed that the prediction of electromagnetic modulation in three narrow bands on the EIT-like spectrum, and a modulation contrast of up to 31 dB was achieved on the transmission through the metamaterial. Our results may facilitate the study on active/dynamical technology in translational metamaterials, which connect extraordinary manipulations on the flow of light in metamaterials, e.g., the exotic EIT, and practical applications in industry.

Metamaterial, a kind of artificial microstructure with unattainable properties in natural occurring medium, has gained wide attention in physics and material science^1^,^2^. The arbitrarily designed optical dielectric-functions of metamaterials have been employed for many novel optical phenomena, such as negative refraction^3^, diffraction-unlimited imaging^4^,^5^,^6^, invisible cloaking^7^, subwavelength cavities^8^,^9^,^10^, and perfect electromagnetic absorber^11^,^12^,^13^,^14^,^15^,^16^ among others. Metamaterials are different from traditional materials for that their responses to external stimuli can be effectively tuned by modeling the geometries of their constituent and thus the local-resonant behaviors. The building blocks of metamaterials, also called meta-atoms, can be rationally designed to mimic the response of natural atoms to external stimuli, which is helpful in realizing classical analogue to quantum phenomena^17^ for potential applications. However, the local-resonant nature of metamaterials limits its operation in a narrow frequency band. It is highly desirable to achieve frequency-agile or multi-band operating metamaterial to extend the working band of a manufactured metamaterial^18^,^19^,^20^,^21^,^22^,^23^,^24^,^25^,^26^,^27^. As part of this development, considerable interest has been focused on the realization of actively controlled metamaterials that exhibit tunable optical response for practical applications in functional optical devices.

Electromagnetically induced transparency (EIT), originates from the quantum interference between different transition pathways within atoms or molecules coupled to laser fields, was suggested as a technique for eliminating the effect of a medium on a propagating beam of electromagnetic radiation, which can be used to remove optical self-focusing and defocusing, and to improve transmission through an opaque medium in a narrow window with low absorption and steep dispersion^28^. The steep dispersion of EIT is promising for slow light and the enhanced light-matter interactions. However, the extreme environment requirement for quantum EIT obstructs it from practical applications in our daily life. Metamaterials provide the opportunities to simply realize classical analogue of quantum phenomena in various frequency band for that their optical properties can be freely tailored by designing the meta-atoms. The spectral characteristics of EIT have been reproduced in several classical structures^29^,^30^,^31^. Especially in recent, artificially structured metamaterials^32^,^33^,^34^,^35^,^36^,^37^,^38^,^39^, were proposed for the demonstration of classical analogue to EIT in plasmonic metamaterial. It is noteworthy that although the classical analogue of EIT in metamaterial (or metamaterial analogue to EIT) show similar spectral response to external stimuli as that in quantum EIT, but the physical mechanism of EIT analogue is different from the quantum EIT in atomic system. In EIT analogue, the EIT-like spectrum is achieved by properly tailoring the coherent interference between resonators, e.g., the EIT analogue was first demonstrated in a metamaterial composed of bright and dark resonators, the two resonators are in close proximity and thus strongly coupled to each other, the coupling or interaction between these two resonator are classical near-field coupling^40^,^41^,^42^,^43^,^44^,^45^, while for the quantum EIT, the coupling between energy levels is implemented through a pump beam^32^.

In this paper, we propose and experimentally demonstrate an electrically controlled metamaterial for multi-frequency electromagnetic modulating^19^. We employed a metamaterial comprising of a single wire coupled to a paired wires, a PIN diode acting as tunable medium is loaded in the gap of the paired wires. The physical mechanism of the metamaterial modulator is to manipulate EIT-like spectrum of the metamaterial by electrically controlling the coupling between the electric mode of a single wire and the magnetic mode of the paired wires. The electrically controlled electromagnetic coupling is implemented by tuning the “on/off” state and the resonant strength of the magnetic mode of the paired wires. It was found both theoretically and experimentally that the proposed metamaterial can serve as a modulator for electromagnetic waves at three discrete bands located around characteristic peak/dips of the EIT-like spectrum. Our results may lead to practical applications based on the electrically controlled electromagnetically induced transparency or other classic analogues to quantum interference phenomena in coherent media^17^.

## Results and Discussion

**Figure 1:**
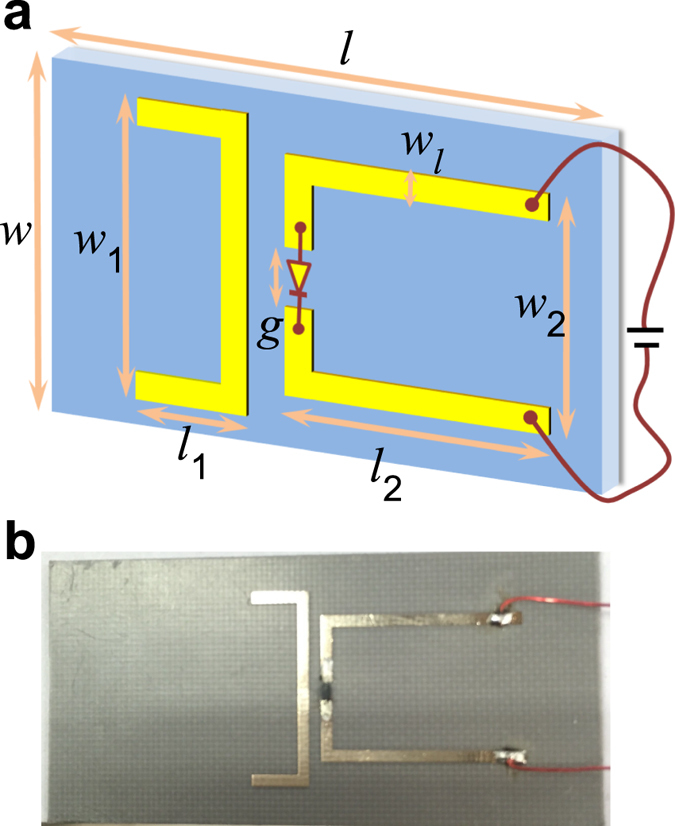
(**a**) Schematic illustration of the proposed metamaterial switch composed of a single wire (left) coupled with a wire pair (right), a PIN diode is located in the gap of the wire pair for active modulation, geometric parameters of the metamaterial are denoted with black letters. (**b**) Photograph of a sample fabricated for experimental measurement.

The schematic of the proposed metamaterial is shown in Fig. 1a, the metallic structure is positioned on a 1 mm-thick dielectric substrate with relative electric permittivity of 2.65. A 35 *μ*m-thick copper film is patterned to metamaterial design with EIT-like spectral response, the structure is composed of a single warped wire (left) coupled with a paired warped wires (right). The width and length of the substrate are w = 22.14 mm and 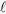 = 47.54 mm, and the width and length of the left wire are *w*_1_ = 16 mm and 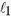 = 5 mm, the width and length of the right wires are *w*_2_ = 12 mm and 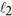 = 17 mm, the metal wires are all with line-width of 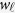 = 1 mm, a gap of g = 1 mm between the paired wires is designed for the loading of a PIN-diode (model: SMP1345).

**Figure 2:**
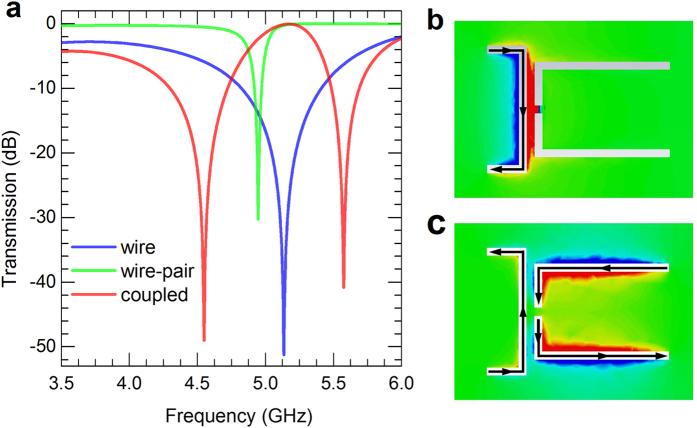
(**a**) Calculated transmission (in dB) spectra of a single warped wire (blue solid), a pair of warped wires (green solid), and the metamaterial with coupled single wire and wire pair (red solid). The distributions of the out-of-plane magnetic fields (black arrows illustrate the surface currents) at the frequency of 5.17 GHz are plotted for a metamaterial composed of a single wire and a wire pair with lumped resistance of 10 Ω (**b**), 7000 Ω (**c**).

We first consider the spectral response of the coupled metamaterial design, the calculations were carried out within a PEC surrounded air box (to simulate our experimental setup: a C-band waveguide). For a metamaterial with only the left wire, it is shown that there is a resonant dip around 5.14 GHz on the transmission spectrum [see in Fig. 2a], the resonance is a broad electric-dipolar mode from our investigation on the local-fields. The dipolar mode is similar to the bright mode of the wire in the plasmonic induced transparency (PIT) study^32^, which provides an opaque spectral range for the formation of EIT analogue. Then we consider the property of the paired wires, the transmission spectrum is presented by the green curve in Fig. 2a, in which a sharp resonance around 4.95 GHz is observed, the sharp resonance was verified to be a magnetic-dipolar mode from the local-field investigations (not presented here). Finally, we simulated the case of a metamaterial comprised of both the left single wire and the right wire pair, the transmission spectrum is presented in Fig. 2a, it can be seen that the original transmission dip of a wire is now changed to a transmission peak at the same frequency with the introduction of the wire pair, and the transparency is accompanied by two transmission dips on either side of the peak, these peak/dips are just the physical picture of constructive and destructive interferences in EIT.

**Figure 3:**
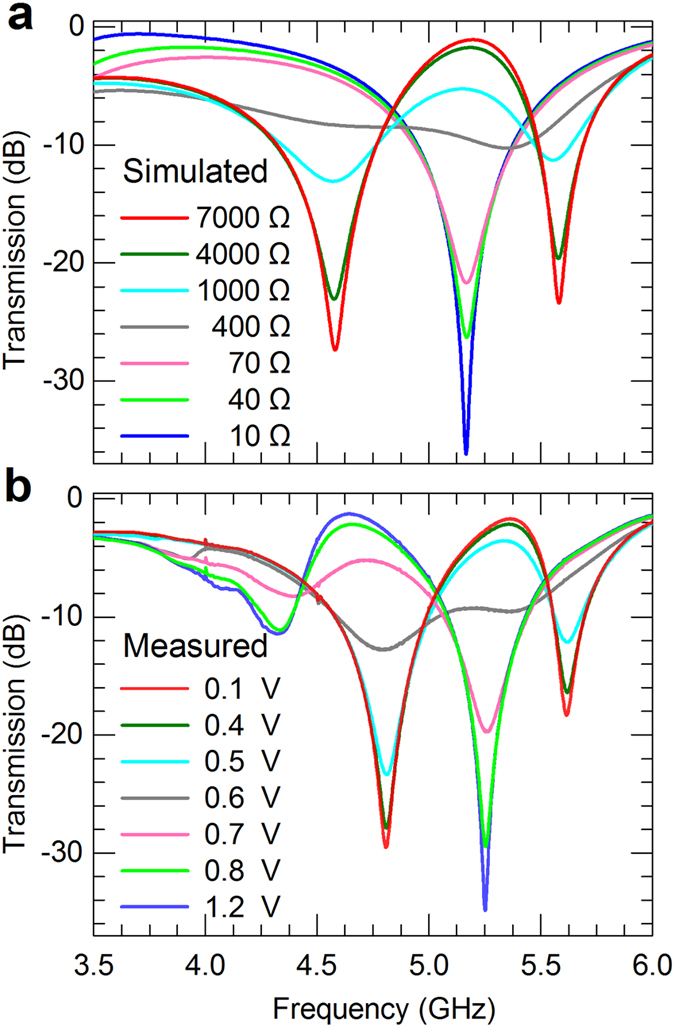
Conductivity dependence of the transport through a metamaterial switch. (**a**) Plot of the simulated transmission spectra (in dB) for a serious of lumped resistance (from 7000 Ω to 10 Ω). (**b**) Measured transmission of the metamaterial switch sample under different biasing voltage changing from 0.1 V to 1.2 V.

It is known that the EIT-like spectrum results from the coherent interference between a sharp resonant and relatively broad resonant background mode, and the sharp resonance is crucial in the formation process of EIT analogue which should be designed: (i) with sharp phase changing compared to the electric resonant background for simultaneously achieving destructive and constructive interferences in a narrow frequency range; (ii) of proper spectral-overlap with respect to the resonance of electric mode of the single wire. In this study, we propose to realize an electrically biased metamaterial modulator at multi-frequencies by taking advantages from the second recipe and the multi-peak/dips EIT-like spectrum. Specifically, we suggest to electrically tuning the conductivity in the gap of paired wires (see Fig. 1) to inductively tune the resonance within the resonant regime of the left single wire. Our numerical results indicate that the magnetic resonance of the wire pair can be excited near the resonant frequency of the electric mode (of the left single wire) by connecting the two discrete wires metallically (results are not shown here), then these two modes will coherently interfere with each other and show EIT-like transmission spectrum. The formation of EIT analogue can thus be interdicted consequently for realizing electromagnetic modulator at specific frequencies. Figure 3a present the simulated transmission spectra (in dB) of different inductance between the paired wires, that is a serious of lumped resistance in the gap, the resistance was changed from 7000 Ω(unconnected) to 10 Ω(connected). As shown in Fig. 3a, the initial EIT-like spectrum for high resistance gradually changes to resonant spectrum of the electric wire with the resistance decreasing to small values like 70 Ω, 40 Ω, and 10 Ω. To get a better understanding of the change of the spectra, we plot in Fig. 2b and c the distributions of the out-of-plane magnetic fields (black arrows illustrate the surface currents) at the frequency of 5.17 GHz for a metamaterial composed of a single wire and a wire pair. For the nearly connected case (Fig. 2b, 10 Ω), only the electric mode of the single wire is excited which induced a transmission dip on the spectrum; while for the unconnected case (Fig. 2c, 7000 Ω), the magnetic mode of the wire pair is well excited through near-coupling with the electric mode of the left single wire, and the coherent interference between these two modes show the EIT analogue on the transmission spectrum, and the transmission changes at the characteristic frequencies (peak/dips) of the EIT-like spectrum will be used for modulating the propagation of electromagnetic waves. The peak around 5.17 GHz vanished and varied to a transmission dip of about −35 dB. The two resonant dips associated with the peak around 4.6 and 5.6 GHz also vanished and varied to be of high transmission. These significant modifications on the spectra at three discrete narrow bands are promising for high efficiency electromagnetic modulating application.

To realize inductive control of the EIT-like spectrum, here we employ a PIN diode to implement the electrically tunable connection of the paired wires, a PIN-diode was inserted between the pair wires. As we know, the diode will be under a high resistance state when it is not biased or the voltage is small, because the P-N junction blocks the flow of the carriers. When the bias voltage increases to some certain level, the drag ability on carriers of P-N junction decreases remarkably and the diode becomes of low resistance. The change of resistance of the PIN diode under biasing is employed to switch the magnetic mode around the resonant frequency of the electric mode of the single wire, which is essential in controlling the EIT-like spectrum as shown in Figs 2 and 3a. The photograph of a sample fabricated for experimental investigation is presented in Fig. 1b, two metallic conductors are welded on the magnetic wire pair for electrically biasing the PIN diode. All the experiments were done in a standard C-band waveguide (model: WR-187), the scattering parameters measurement were carried out using a vector network analyzer (model: AV3629D). In the experiment, the diode was connected to an accurately tunable source to control the bias voltage.

Figure 3b presents the measured transmission of the metamaterial sample under different bias voltages changing from 0.1 V to 1.2 V. For not biased or low bias voltages situations, for example the bias voltage of 0.1 V, the transmission spectrum shows a peak around 5.25 GHz. Then we increased the bias voltage on the diode, it can be seen that the peak around 5.25 GHz changes dramatically to a transmission dip of about −35 dB during the bias voltage increasing from 0.1 V to the 1.2 V. The measured electric switch behavior at the EIT-like peak frequency agrees well with our theoretical prediction as presented in Fig. 3a (in which the values of resistance were chosen to compare with the measured spectra), indicating that the inductive modification on the magnetic resonance of the wire pair can be used for practical modulating application. We also notice that the two dips associated with the peak vanished gradually as the bias voltage increasing as the calculated results. However, an additional dip appears on the transmission spectrum around 4.4 GHz for higher bias voltages like 0.7 V, 0.8 V, and 1.2 V. We confirmed that the unexpected dip is due to the introduction of the biasing arms [red wires in Fig. 1b], the biasing arms extended the size of paired wires and thus shifted the magnetic resonance of connected wire pair to about 4.4 GHz (the paired wires are almost electrically connected under high biasing voltages, and thus the resistance of the diode is rather low), the biasing arms also make the measured working frequencies are higher than the prediction without considering the biasing. To make a short summary, although the measured results are slightly different from the predicted results [see Fig. 3a] of the numerical simulations which is due to the biasing in measurements, our experiments generally validated the feasibility of our proposal that the novel EIT phenomenon can be controlled to realize metamaterial modulators at a subwavelength scale.

**Figure 4:**
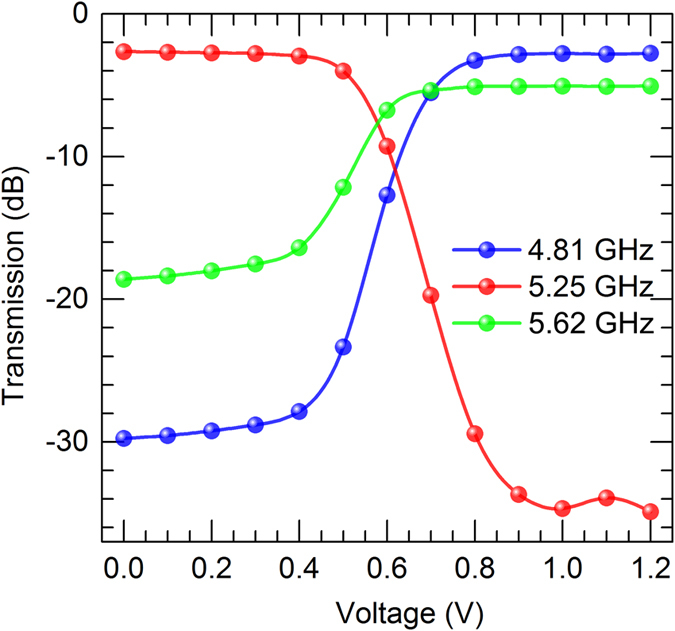
Measured transmission (in dB) versus biasing voltage on the diode (from 0 to 1.2 V) at the three switching frequencies. The transmission are plotted for the EIT-like peak frequency 5.25 GHz (red), EIT-like dip frequencies 4.81 GHz (blue) and 5.62 GHz (green).

To show more details on the working performance of our metamaterial modulator, we plot the measured transmission at three characteristic frequencies of the EIT-like spectrum in Fig. 4. Remarkable modulation in transmission can be achieved through electric biasing, that transmission at 5.25 GHz, 4.81 GHz, and 5.62 GHz. The modulation curve at 5.25 GHz, shown as red curve in Fig. 4, shows a range with bias voltage from 0.4 V to 0.9 V, during which the transmission drops rapidly from the high transmission region to low transmission region with modulation ratio of about 31 dB. The modulations at 4.81 GHz and 5.62 GHz show changing in the same bias voltage range, and the transmission rises from low transmission level to high transmission level as the bias voltage increases, with modulation ratio of about 27 dB and 14 dB, respectively. Generally speaking, we find that the transmission can be greatly modulated under a small change in the bias voltage at multiple frequencies thanks to the novel EIT-like spectrum and the sensitive tunability of the magnetic resonance of the wire pair.

## Conclusions

In conclusion, we experimentally demonstrated an electrically controlled metamaterial modulator by tuning the formation process of EIT-like spectrum. Since that the EIT spectrum comes from the coherent interference of a sharp resonance with a background of broaden response, we proposed to control the EIT-like spectrum by introducing a PIN diode between a pair of wires, the resistance of the diode can be electrically tuned for inductively controlling the magnetic mode of the paired wires and thus the transmission spectrum. As a consequence, the transmission of electromagnetic waves in the three characteristic bands on the EIT-like spectrum can be effectively modulated by the voltages on the diode. The principle experiments confirmed that the transmission of the metamaterial switch can be significantly modulated under rather small biasing voltages at the characteristic peak/dips of EIT-like spectrum and the multi-frequency modulating based on electrically controllable EIT analogue has benefits for extending the working band of metamaterial based resonant modulators^46^.

## Methods

The numerical results were obtained with a Finite-Difference-Time-Domain (FDTD) based electromagnetic solver. The perfect E boundary conditions were set for the simulation. All the experiments were done in a standard C-band waveguide (model: WR-187) and the scattering parameters were recorded using a vector network analyzer (model: AV3629D).

## Additional Information

**How to cite this article**: Fan, Y. *et al*. An electromagnetic modulator based on electrically controllable metamaterial analogue to electromagnetically induced transparency. *Sci. Rep.* **7**, 40441; doi: 10.1038/srep40441 (2017).

**Publisher's note:** Springer Nature remains neutral with regard to jurisdictional claims in published maps and institutional affiliations.
